# Molecular Alterations in Pediatric Sarcomas: Potential Targets for Immunotherapy

**DOI:** 10.1080/13577149878037

**Published:** 1998-06

**Authors:** Theresa J. Goletz, Crystal L. Mackall, Jay A. Berzofsky, Lee J. Helman

**Affiliations:** 1 Molecular Immunogenetics and Vaccine Research Section Metabolism Branch National Cancer Institute National Institutes of Health Bethesda MD 20892 USA; 2 Molecular Oncology Section, Pediatric Branch Division of Clinical Sciences, National Cancer Institute National Institutes of Health, Building 10 Room 13N240, 9000 Rockville Pike Bethesda MD 20892–1928 USA

## Abstract

*Purpose/results/discussion.* Recurrent chromosomal translocations are common features of many human malignancies. While such translocations often serve as diagnostic markers, molecular analysis of these breakpoint regions and the characterization of the affected genes is leading to a greater understanding of the causal role such translocations play in malignant transformation. A common theme that is emerging from the study of tumor-associated translocations is the generation of chimeric genes that, when expressed, frequently retain many of the functional properties of the wild-type genes from which they originated. Sarcomas, in particular, harbor chimeric genes that are often derived from transcription factors, suggesting that the resulting chimeric transcription factors contribute to tumorigenesis. The tumor-specific expression of the fusion proteins make them likely candidates for tumor-associated antigens (TAA) and are thus of interest in the development of new therapies. The focus of this review will be on the translocation events associated with Ewing's sarcomas/PNETs (ES), alveolar rhabdomyosarcoma (ARMS), malignant melanoma of soft parts (MMSP) (clear cell sarcoma), desmoplastic small round cell tumor (DSRCT), synovial sarcoma (SS), and liposarcoma (LS), and the potential for targeting the resulting chimeric proteins in novel immunotherapies.


*Sarcoma* (1998) **2**, 77–87

REVIEW

### Molecular alterations in pediatric sarcomas: potential targets for immunotherapy

THERESA J. GOLETZ,[1] CRYSTAL L. MACKALL,[2] JAY A. BERZOFSKY[1] & LEE J. HELMAN[2]

[1]*Molecular Immunogenetics and Vaccine Research Section, Metabolism Branch &* [2]*Molecular Oncology Section, Pediatric Oncology Branch, National Cancer Institute, National Institutes of Health, Bethesda, MD 20892, USA*

**Abstract**

*Purpose/results/discussion.* Recurrent chromosomal translocations are common features of many human malignancies. While such translocations often serve as diagnostic markers, molecular analysis of these breakpoint regions and the characterization of the affected genes is leading to a greater understanding of the causal role such translocations play in malignant transformation. A common theme that is emerging from the study of tumor-associated translocations is the generation of chimeric genes that, when expressed, frequently retain many of the functional properties of the wild-type genes from which they originated. Sarcomas, in particular, harbor chimeric genes that are often derived from transcription factors, suggesting that the resulting chimeric transcription factors contribute to tumorigenesis. The tumor-specific expression of the fusion proteins make them likely candidates for tumor-associated antigens (TAA) and are thus of interest in the development of new therapies. The focus of this review will be on the translocation events associated with Ewing's sarcomas/PNETs (ES), alveolar rhabdomyosarcoma (ARMS), malignant melanoma of soft parts (MMSP) (clear cell sarcoma), desmoplastic small round cell tumor (DSRCT), synovial sarcoma (SS), and liposarcoma (LS), and the potential for targeting the resulting chimeric proteins in novel immunotherapies.

### Introduction

Chromosomal abnormalities are common in human tumors with many malignancies exhibiting clonal chromosomal aberrations.[1] The identification of tumor-specific chromosomal translocations aids in diagnosis and serves as a prognostic indicator.[2–6] With an increasing understanding of the effect these events have on normal cellular processes, novel therapies can be developed which have greater specificity and efficacy.

Two major consequences of chromosomal rearrangements in tumors have been identified: the activation of an oncogene, or the creation of a novel oncogenic protein. First, translocations can result in the activation of genes located at or near the breakpoint. Often, these genes normally function in the promotion of cell growth and differentiation. Thus, their disruption can affect normal cell regulation. This type of alteration, which is most common in hematological malignancies, is illustrated by the t(8;14) translocation associated with Burkitt's lymphoma in which *c-MYC* is activated by repositioning under the control of the potent Ig enhancer.[1] An alternative consequence of chromosomal translocations is the generation of functional chimeric genes. This scenario is most common in solid tumors and usually involves unrelated genes. Often, these translocation events affect genes encoding transcription factors, thereby generating chimeric transcription factors with properties of both genes (Table 1). The fusion proteins often exhibit the DNA-binding specificity of one gene with the activation domain of the other gene. Such fusion proteins activate/repress transcription, exhibit altered DNA binding specificity or participate in novel protein–protein interactions. Thus, they are thought to play a critical role in the neoplastic transformation process.

The identification of translocations associated with a group of primitive sarcomas, and the subsequent cloning of the chromosomal breakpoint regions, has revealed that a common theme in these tumors is the generation of chimeric transcription factors. The fusion proteins are expressed exclusively in the tumor cells, and function as potent transcription factors where they are thought to contribute to neoplastic transformation by mediating

Correspondence to: L. J. Helman, Molecular Oncology Section, Pediatric Branch, Division of Clinical Sciences, National Cancer Institute, National Institutes of Health, Building 10, Room 13N240, 9000 Rockville Pike, Bethesda, MD 20892–1928, USA. Tel: +1 301 4964257; Fax: +1 301 4020575; E-mail: helmanl@pbmac.nci.nih.gov.

1357-714X/98/020077-11 ©1998 Carfax Publishing Ltd

**Table 1**. *Tumor-specific translocations associated with solid tumors*

| Tumor | Translocation | 5′/3′ fusion product | Type |
|---|---|---|---|
| Ewing's sarcoma/ PNET | t(11;22)(q24;q12)<br>t(21;22)(q22;q12)<br>t(7;22)(p22;q12) | EWS/FLI-1<br>EWS/ERG<br>EWS/ETV1 | RNA binding<br>ETS TF |
| Alveolar rhabdomyosarcoma | t(2;13)(q35;q14)<br>t(1;13)(p36;q14) | PAX3/FKHR<br>PAX7/FKHR | PB and HD/FD |
| Melanoma of soft parts (clear cell sarcoma) | t(12;22)(q13;q12) | EWS/ATF1 | RNA binding/ bZIP TF |
| DSRCT | t(11;22)(p13;q12) | EWS/WT1 | RNA binding/ Zn finger TF |
| Synovial sarcoma | t(X;18)(p11.2;q11.2) | SYT/SSX1<br>SYT/SSX2 | SH2/KRAB box |
| Liposarcoma (myxoid and round cell) | t(12:16)(q13;p11) | CHOP/FUS-TLS | RNA binding/ bZip TF |

aberrant expression of normal genes. Several of the chimeric genes have been cloned and found to confer a transformed phenotype when expressed *in vitro*.[7–11] The tumor-specific expression of the fusion proteins make them likely candidates for tumor-associated antigens (TAA), in which the junction point creates a neo-antigenic determinant. The focus of this review will be on the translocation events associated with Ewing's sarcomas/primitive neuroectodermal tumors (PNETs) (ES), alveolar rhabdomyosarcoma (ARMS), malignant melanoma of soft parts (MMSP or clear cell sarcoma), desmoplastic small round cell tumor (DSRCT), synovial sarcoma (SS), and liposarcoma (LS), and the potential for targeting the resulting chimeric proteins in novel immunotherapies.

### Tumor-associated chromosomal translocations in pediatric sarcomas

### *Ewing's sarcoma/primitive neuroectodermal tumors*

The ES/PNET family of tumors is a group of poorly differentiated malignancies that include Ewing's sarcoma (ES), peripheral neuroepithelioma (PNET) and Askin's tumor. They are thought to originate from the neuroectoderm, and show varying, but limited degrees of neural differentiation. These tumors express MIC2, a membrane protein that appears to function in cellular adhesion. The expression of this antigen distinguishes these tumors from other small round cell malignancies.[12,13] In addition, approximately 85% of ES/PNET tumors are characterized by t(11;22)(q24;q12).[14–17] Delattre *et al.* demonstrated that the t(11;22)(q24;q12) rearranges the *FLI1* gene (Friend leukemia integration site 1) on chromosome 11q24 with a heretofore uncharacterized gene, *EWS*.[8,18] There is no evidence for the expression of the reciprocal hybrid transcript.[19]

*EWS* encodes a 656-aa protein, the function of which remains unclear. While this protein is ubiquitously expressed, expression levels fluctuate with the cell cycle.[19–23] *EWS* contains two major functional domains. The first is the N-terminal region (exons 1–7) consisting of a series of degenerate repeats that resemble the transactivation domains of several transcription factors, such as SP-1[24] while the second region, the C-terminal region, includes a putative RNA-binding domain (exons 11–13) defined by a conserved 80-aa domain.[24] Wild-type EWS has been shown to bind RNA *in vitro* and EWS/GAL4 fusion proteins can activate a reporter gene, suggesting a role for EWS in transcription.[9,21,23]

FLI1, a member of the ETS family of transcription factors, is the human homologue of the murine *FLI1* gene and is normally expressed in hematopoietic tissues.[25] The ETS DNA-binding domain, usually located in the C-terminal portion of the protein, is an 85-aa region that recognizes target genes through a conserved GGAA/T sequence.[26] In *FLI1*, the ETS domain is encoded in the C-terminus, and the N-terminal region contains a domain that is functional in reporter gene assays.[9,27]

EWS/FLI1 is a potent transcription factor that can transform NIH 3T3 cells, and studies have shown that sequences in both EWS and FLI1 are essential for transformation.[7–9] To better define the functional regions of the fusion protein, substitutions were made in which domain 1 of EWS was replaced with a strong heterologous activation domain. Many of these fusion proteins retained activity, although not all were transforming.[7,23] Domain 2 of EWS could also be exchanged with a weak transcriptional activation domain from TLS/FUS without loss of activity. Thus, these data support a model wherein the EWS region of EWS/FLI1 confers strong transactivation through domain 1 with additional properties (protein–protein interaction) contributed by domain 2.

Several variants of the t(11;22)(q24;q12) EWS/FLI1 gene fusion have been described,[8,19] but most include *EWS* exons 1–7 and *FLI 1* exons 8 and 9.[2,28] Therefore, the amino terminal portion of EWS is always fused to the carboxy terminal region of FLI1[8,19] which suggests that these EWS/FLI1 variants contribute to oncogenesis by similar mechanisms.

EWS/FLI1 and FLI1 have similar DNA-binding specificity and affinities,[9,29] but EWS/FLI1 is a more potent transactivator than FLI1.[9,29,30] *In vitro* studies suggested that EWS/FLI1 functioned as a transactivator at 10-fold lower concentrations than FLI1.[29] Thus, it is likely that EWS/FLI1 mediates its transforming effects, at least in part, by transactivation of FLI1 targets or promoters containing ETS-binding sites. Because *c-MYC* is upregulated in some tumors, including ES, one potential target gene of EWS/FLI1 was thought to be *c-MYC*. A study by Bailly *et al.* investigated transactivation of *c-MYC* by EWS/FLI1 using transient tranfection HeLa cells. These experiments suggested that EWS/FLI1 played a role in increased expression of *c-MYC*. However, direct binding of EWS/FLI1 to ETS-binding sites in the *c-MYC* promoter could not be detected using gel shift mobility assays. Thus, EWS/FLI1 upregulates *c-MYC*, albeit by an indirect mechanism yet to be elucidated.[29]

Recent studies suggest that EWS/FLI and FLI1 exhibit some differences in DNA-binding and protein–protein interactions.[31] Therefore, it is possible that EWS/FLI1 also contributes to transformation by activating genes not normally regulated by FLI1. Studies are ongoing to identify the normal targets of EWS/FLI1 and FLI1. Braun *et al.*[32] utilized representational difference analysis (RDA) to identify differentially expressed genes from NIH 3T3 cells containing EWS/FLI1 or normal FLI1. This approach revealed that several transcripts were dependent on the fusion protein for expression, while at least two transcripts were repressed. Stromelysin 1, cytokeratin 15, and a murine homolog of cytochrome P-450 F1 are all induced following expression of EWS/FLI1. However, the kinetics of expression argue against the direct upregulation of all of these target genes. The elucidation of such primary targets will provide insight into the role of EWS/FLI1 in transformation. It is likely that the oncogenic properties of EWS/FLI1 results from both the inappropriate expression of FLI1 target genes, as well as novel protein–protein interactions which may lead to the activation of non-FLI1 target genes. Studies that utilized antisense EWS/FLI1 cDNA to diminish EWS/FLI1 RNA levels demonstrated markedly decreased cell growth in vitro, thereby implicating the fusion protein as a key contributor to aberrant growth.[33,34] EWS/FLI1 may contribute to oncogenesis is by inhibition or alteration of normal apoptotic pathways. Yi *et al.*[35] observed suppression of apoptosis in Ewing's sarcoma cells expressing EWS/FLI1 and found that expression of the fusion protein antisense RNA increased susceptibility to apoptosis. Thus, EWS/FLI1 may contribute to malignant transformation by alteration of more than one gene or gene pathways.

The *EWS* gene is also involved in several other tumor-associated translocations. For example, a minority of PNETs present with a variant t(21; 22) translocation that fuses *EWS* to the *ERG* gene.[3,28,36] Like FLI1, ERG is a member of the ETS family of transcription factors and may regulate similar target genes.[32] Studies are underway to identify ERG target genes. Several lines of evidence suggest EWS/ERG may contribute to neoplastic transformation by the same or similar mechanisms as EWS/FLI1. First, PNETs containing EWS/FLI1 or EWS/ERG are phenotypically and clinically indistinguishable.[2,36] As is seen in EWS/FLI1, EWS/ERG fusions include *EWS* exons 1–7, with *ERG* sequences encoding the ETS domain.[3,28,36] The fusion protein also functions as a transcription factor and requires the same regions for transactivation defined in EWS/FLI1 studies.[21] Furthermore, cells expressing EWS/ERG have a decreased ability to undergo apoptosis. These cells could be made susceptible to apoptosis by the expression of EWS/ERG antisense RNA.[35] Therefore, it is likely that EWS/ERG fusions contribute to oncogenesis in a manner similar to EWS/FLI1.

A rare, third variant, t(7:22)(p22;q12) has been described[37] in which *EWS* is fused to *ETVI,* the human homolog of the murine ETS gene *ER81*. It is likely that EWS/ETV1 contributes to malignant transformation by mediating aberrant transcription and/or repressing expression of regulatory genes. However, RDA analysis of EWS/ETV1 revealed that only one of eight EWS/FLI1 target genes was upregulated by EWS/ETV1. This suggests that EWS/ETV1 activates only a portion of the EWS/FLI1 transformation pathway, requiring other alterations for tumorigenesis, or that EWS/ETV1 plays a minor role in transformation. Further studies are needed to define the effect of EWS/ETV1 on normal gene expression.

Recently, Peter *et al.* identified a new member of the ETS family fused to *EWS* in Ewing's sarcoma, the *FEV* gene.[38] *FEV,* which maps to chromosome 2, encodes a 238-aa protein. Its expression is highly restricted with protein being detected only in adult prostate and small intestines, but not in other fetal or adult tissues. FEV contains an ETS DNA binding domain closely related to that of ERG and FLI1; however, in contrast to these proteins, FEV has a small N-terminal region of only 42 aa which suggests that it lacks important transcription regulatory domains present in other ETS family proteins. It is unclear whether or not EWS/FEV alters transcription of similar target genes than other EWS fusion proteins. Further studies are needed to elucidate this fusion protein's role in the pathogenesis of ES.

The common denominator of these tumors is that all are primitive neuroectodermal sarcomas occurring in children and young adults, and the evidence strongly implicates EWS fusions as key mediators of malignant transformation. There is also strong evidence to suggest that these fusion proteins contribute to oncogenesis by aberrant expression of target genes (activation and repression), as well as altering the expression of genes not normally regulated by the native transcription factors.[32] Furthermore, these genes may effect normal growth regulation by interfering with apoptotic pathways.[35]

### *Alveolar rhabdomyosarcoma (ARMS)*

Rhabdomyosarcoma is the most common soft tissue sarcoma in pediatric patients, with approximately 250 cases per year in the United States. Roughly 20% of these cases are of the alveolar morphological type (ARMS) which is characterized by alveolar-like spaces formed by fibrovascular septa. These spaces are filled with malignant cells that are distinguished by their eosinophilic cytoplasm. Approximately 80% of ARMS express a translocation involving the long arms of chromosomes 2 and 13 t(2;13)(q35;q14), which results in the juxtapositioning of a truncated *PAX3* gene of chromosome 2 to the 3′-terminal region of the *FKHR* gene of chromosome 13.[39–43]

The PAX family of transcription factors play important roles during embryonic development, particularly in morphogenesis and pattern formation.[44] These genes contain a paired-box (PB) DNA-binding domain and some also contain a homeobox (HB) DNA-binding domain. Overexpression of these genes can result in oncogenic transformation[10,11] and loss of function mutations has been observed in several genetic diseases, including Waardenburg syndrome.[45]

FKHR, formally known as ALV,[41] is a member of the fork-head domain (FD) family of transcription factors which contain a conserved DNA-binding motif related to the *Drosophila* region-specific homeotic gene *fork-head*. This family of transcription factors normally functions during embryogenesis. The *FKHR* gene is ubiquitously expressed and functions as a transcription factor.

The hybrid gene which results from the t(2;13)(q35;q14) translocation encodes a fusion protein containing the amino terminal portion of the PAX3 protein including the PB and HB domains joined to the carboxyl region of the FKHR protein that is truncated within the winged helix DNA-binding region, but retains a putative transactivation domain. Evidence suggests that the DNA-binding specificty of PAX3/FKHR is contributed by PAX3, most likely through the PB and HB domains, while FKHR contributes the transactivation region. Although the DNA-binding activity of PAX3/FKHR is less than wild-type PAX3, the fusion protein is a more potent transactivator.[46–49] Overexpression of murine PAX3 transforms NIH 3T3 cells[11] and the PAX3/FKHR fusion protein transformed chicken embryo fibroblasts.[10] One possible mechanism of transformation is through a gain of function, not only by increased transactivation potency, but also through constitutive and increased expression.[49,50] Interestingly, a recent study which utilized antisense technology to downregulate PAX3/FKHR in ARMS tumor cells demonstrated reduced cell viability, which led to the conclusion that PAX3/FKHR may contribute to malignant transformation through suppression of apoptotic processes which would normally cause cell death.[51]

Interestingly, 10–20% of ARMS tumors contain a variant translocation, t(1;13)(p36;q14), that results in the in-frame fusion of 5′ PAX7 to 3′ FKHR. PAX7 and PAX3 are highly homologous in the PB and HB domains, suggesting that they might recognize similar target genes.[40–43,52] Furthermore, the PAX3/FKHR and PAX7/FKHR chimeric proteins share structural similarities in that they both contain intact N-terminal PB and HB regions fused to the acidic and proline-rich C-terminal region of FKHR.[41,42,52] Therefore, it is likely that these translocations create similar chimeric transcription factors that contribute to transformation by altering expression of a common group of target genes.[50–52]

### *Malignant melanoma of soft parts (MMSP) or clear cell sarcoma (CCS)*

Malignant melanoma of soft parts (MMSP), also known as clear cell sarcoma (CCS), is a rare, but aggressive soft tissue sarcoma of muscle tendons and aponeuroses that occurs most frequently in young adults between the ages of 15 and 35 years.[53] Over 95% of MMSP cases occur in the extremities, and only rarely (less than 2%) occur in the head and neck region. Although MMSP is a melanin-producing tumor, there is no evidence to suggest that these tumors are directly related to malignant melanoma. MMSP is thought to have neuroectodermal origins[54] and expresses neural antigens, as well as markers of melanin production, such as HMB-45. A t(12;22)(q13;q12) translocation event is present in more than 70% of these tumors[55,56] and molecular analysis of the breakpoint reveals an *EWS/ATF1* fusion. This chimeric protein joins the 5′ RNA-binding region of the *EWS* gene and the 3′ region of the *ATF1* gene, a member of the CREB/transcription factor family of leucine zipper transcription factors that has a bZIP domain for DNA binding and protein–protein interaction.[57] This family of transcription factors mediates transcription through ATF-binding sites. The expression of these genes is induced by cAMP, and they are activated by phosphorylation by cAMP-dependent protein kinase A (PKA).[58,59]

The t(12; 22) translocation fuses the N-terminal portion of *EWS* to the C-terminal region of *ATF1*, retaining the bZIP domain. However, the PKA regulatory phosphorylation site is lost.[58] Thus, it is likely that EWS/ATF1 could exhibit the DNA-binding specificity of ATF1, and dimerize with CREB, but would not be cAMP-inducible. EWS/ATF1 does activate promoters with ATF1 binding sites, although not all such promoters were activated,[60] and some promoters were found to be repressed by EWS/ATF1. Therefore, EWS/ATF1 may contribute to malignant transformation by several mechanisms. First, EWS/ATF1 may constitutively activate ATF1 target genes that are normally induced by cAMP, or it may repress genes that normally function in growth control. Alternatively, EWS/ATF1 may activate novel genes, perhaps genes regulated by other CREB/ATF family members.

In most MMSP tumors, two hybrid transcripts are generated and expressed by the t(12;22)(p13;q12) translocation. The expression profile of the fusion gene on der(12) chromosome is compatible with the ubiquitous expression of ATF. However, this out-of-frame fusion results in a product consisting of the first 65 N-terminal amino acids of ATF1, which is unlikely to bind DNA or dimerize, making its role in transformation unclear. It is unlikely that expression of the der(12) transcript is essential in transformation given reports that 30% of MMSP lack expression.[56]

### *Desmoplastic small round cell tumor (DSRCT)*

Desmoplastic small round cell tumor (DSRCT) is an aggressive small round cell tumor that occurs predominantly in abdominal serosal surfaces and has a predilection for young males.[61] The tumor is a primitive small round cell with features of divergent differentiation, co-expressing epithelial, neural and myogenic markers. The origin of this tumor remains unclear, but it is most likely derived from the mesothelium. Almost 100% of these tumors contain a t(11;22)(p13;q12) translocation that fuses the 5′ region of the *EWS* gene to the 3′ region of *WT1,* a tumor suppressor gene involved in a subset of Wilms' tumors.[62–66] WT1 binds DNA through a series of zinc fingers and represses the transcription of certain genes. These zinc fingers are essential for transcriptional repression. The chimeric protein contains the N-terminal region of EWS fused to the WT1 DNA-binding domain. Given that both the wild-type *EWS* gene and EWS fusion proteins are known to participate in transcriptional complexes, it is likely that EWS/WT1 functions as a transcription factor, possibly through WT1 targets. Therefore, unlike the loss of function mutation in Wilm's tumor, the loss of the zinc finger region of WT1 in EWS/WT1 serves to convert WT1 from a repressor of transcription to a dominant transcriptional activator oncogene.[67]

### *Synovial sarcoma (SS)*

Synovial sarcoma is an aggressive soft-tissue malignancy which occurs primarily in the extremities near major joints (e.g. ankle, knee) of adolescents and young adults. Virtually all synovial sarcomas contain a translocation of chromosomes X and 18[68] with approximately 70% involving t(X;18)(p11.2;q11.2). This translocation event generates a fusion protein from the 5′ region of the *SYT* gene and the 3′ region of *SSX1* or *SSX2*.[69–71] There is no evidence of a transcript being expressed by the reciprical hybrid der (18).[71] The function of the *SYT* gene is unknown, and sequence analysis reveals no classical structural motifs associated with DNA-binding or transcriptional regulation. However, the presence of SH2 and SH3 domains suggests that SYT might function through protein–protein interaction. The recent isolation of the mouse homolog of SYT revealed that SYT is expressed ubiquitously during early embryogenesis,[69] but expression is restricted later in development to cartilage tissue, specific neuronal cells and some epithelial-derived tissues. SYT was also detectable in primary spermatocytes.

Several studies suggested that SS contained two distinct X chromosome breakpoint sites. However, the identification of two closely related genes at Xp11.2 established the involvement of distinct coding regions. Despite being 2 Mb apart, *SSX1* and *SSX2* share 80% homology.[70] Both encode a 188-aa protein with an N-terminal Kruppel-associated box (KRAB) that is thought to function as a transcription repressor domain.[72,73] Although these proteins lack zinc finger motifs, the presence of the KRAB sequences suggest a role in transcription. However, this domain is not present in the chimeric protein, which suggests that SSX1 and SSX2 sequences contribute to transformation through novel protein–protein interactions or some other function. SSX3, another KRAB protein, is not implicated in t(X;18)-positive SS,[74] but has high homology to SSX1 and SSX2 (95 and 90%, respectively). The study of this gene may provide insight into the function of SSX1 and SSX2.

### *Liposarcomas (LPS)*

Liposarcomas (LS) are soft tissue tumors that occur primarily in the extremities and retroperitoneum. These tumors are from primitive mesenchymal cells and they resemble fetal adipose tissue. Several characteristic cytogenetic aberrations have been identified for adipose tumors. The most common LS are myxoid round cell liposarcomas, and greater than 90% of myxoid liposarcomas contain the t(12;16)(q13;p11) translocation in which *CHOP* on the long arm of chromosome 12 is fused to *FUS/TLS*.[22,75–77] However, this translocation event has not been detected in other adipose tumors and, therefore, may provide interesting insight into the transformation process of this subset of tumors.

FUS/TLS is structurally similar to EWS (>50% amino acid identity)[75] and is expressed at high levels in all tissues examined.[22] TLS binds RNA and encodes a strong transcriptional activation domain in the N-terminal region.[78] Therefore, like EWS, FUS/TLS may function as a nuclear RNA-binding protein.

CHOP, also called GADD153, is a member of the CCATT/enhancer-binding protein (C/EBP) family of leucine zipper transcription factors that regulate adipocyte differentiation. CHOP is expressed at low levels in adipocytes; however, mRNA levels increase during conditions of stress such as DNA damage. Overexpression of CHOP in NIH 3T3 cells results in growth arrest at G1/S.[79] Thus, CHOP is thought to function as a dominant negative growth regulator.[80]

In the TLS/CHOP fusion protein, the N-terminal portion of TLS is joined to the entire CHOP coding region.[75,76] TLS/CHOP can transform NIH 3T3 cells and studies indicate that transformation requires sequences from both TLS and CHOP.[78] The requirement for the C-terminal leucine zipper domain of CHOP for transformation suggests a crucial role for C/EBP protein dimerization. Although it is unclear whether normal wild-type CHOP activation requires DNA-binding, the potential DNA-binding region, a basic region of the bZIP domain, is required for transformation. The role of TLS sequences in transformation may be more than that of a strong transactivator, since substitution of this region with other potent transactivating domains did not mediate transformation. However, substitutions with EWS sequences were transforming.[78] Therefore, TLS/CHOP may contribute to transformation by mechanisms similar to those previously discussed in EWS fusion proteins.

### Potential immunotherapeutic approaches for the treatment of pediatric sarcomas

Although multi-modality therapy has improved survival rates for the pediatric sarcomas described in this review, patients often relapse, at which time responses to multi-agent chemotherapy are brief or non-existent. Furthermore, patients who present with metastatic disease at diagnosis do very poorly in spite of aggressive multi-modality therapy. Therefore, efforts are needed to develop novel treatments, such as immunotherapies. Studies over the past decade have provided evidence that treatments based on the manipulation of the immune system can mediate regression of established metastatic cancer. More specifically, cell-mediated immunity can play a critical role in tumor regression.

T lymphocytes are most often categorized as $CD8^+$ cytotoxic lymphocytes (CTL) or $CD4^+$ helper lymphocytes (Th), and both types of T cells are known to play a role in tumor regression. Our understanding of antigen processing, presentation, and recognition has increased considerably in the last two decades and has been expertly reviewed elsewhere.[81] Briefly, T cells recognize antigens as short peptides that are bound to the cell surface in the context of major histocompatibility (MHC) molecules.[81,82] In the case of $CD8^+$ CTL, the T cell receptor (TCR) recognizes short peptides (8–10 amino acids) bound to MHC class I molecules. These peptides are derived from endogenously expressed proteins which undergo proteolytic processing in the cytosol by large proteosome complexes. Peptide fragments are then transported into the lumen of the endoplasmic reticulum (ER) by specialized transporters of antigen processing (TAP). Once inside the ER, peptides associate with an appropriate MHC class I molecule that is associated with beta-2-microglobulin ($\beta$2 m), an invariant subunit which is thought to enhance efficient MHC folding, optimize MHC/peptide binding, and increase stability of the MHC/peptide complex during transport to and expression on the cell surface. Following peptide/MHC binding, the peptide/MHC/$\beta$2 m complexes transverse the ER and Golgi apparatus, and are displayed on the cell's surface where they are subject to surveillance by CTL. In the case of $CD4^+$ Th cells, the TCR recognize slightly larger peptides (10–25 aa) in the context of MHC class II molecules. These peptides are typically derived from material or organisms which have undergone endo/phagocytosis by APC. Thus, in general, $CD8^+$ CTL recognize intracellular (endogenous) peptides while $CD4^+$ T cells recognize external (exogenous) protein fragments.

CTL can distinguish self from non-self peptides associated with MHC class I molecules, so that expression of viral proteins or altered cellular proteins will be reflected in the peptide/MHC complexes displayed on the cell surface. Although the tumor-specific fusion proteins described in this review function as nuclear transcription factors, they are still subject to the proteolytic processing and presentation pathways described. There is experimental evidence that tumor-associated nuclear proteins, such as mutant p53, can induce immune responses.[83–88].

The identification of TAA and an increased understanding of the requirements for the induction of cell-mediated immune responses (Table 2) has led to advances in immunotherapy.[89] While a number of TAA have been identified for several tumor types,[90–93] it is unclear whether all TAA will be effective tumor regression antigens. Ideally, one would like to identify and target TAA which play a key role in neoplastic transformation, so that they cannot be lost without loss of malignancy. The tumor-associated translocations identified for a number of pediatric sarcomas such as ES and AR may very well be such antigens, since they generate functional chimeric transcription factors known to contribute to abberrant gene expression. More

**Table 2**. *Immunotherapeutic approaches using tumor-associated antigens*

| |
|---|
| Active immunotherapy using immunodominant peptides: |
| alone |
| with adjuvants |
| linked to helper peptides |
| Administered: |
| in lipids/liposomes |
| pulsed onto antigen-presenting cells (APCs) |
| Substituted peptides |
| immunodominant peptides with amino acid substitutions to increase binding to MHC |
| Proteins |
| alone |
| with adjuvants |
| DNA |
| 'naked' DNA encoding cancer antigens administered using gene gun |
| intramuscular injection |
| associated/linked to lipids |
| Recombinant viruses |
| recombinant viruses, such as vaccinia, fowlpox or adenovirus, encoding cancer antigens, alone or in combination with genes encoding cytokines costimulatory molecules or immunostimulatory factors |
| Recombinant bacteria |
| recombinant bacteria such as bacillus calmette–guerin (BCG), *Salmonella* or *Listeria* engineered to express cancer antigens alone or with genes encoding cytokines, costimulatory molecules or other immunostimulatory factors |
| Active immunotherapy followed by cytokines |
| Interleukin 2 (IL-2), IL-6, IL-10, IL-15 |
| Passive immunotherapy with anti-tumor lymphocytes generated *in vitro* |
| Generation of CTL using immunodominant peptide-pulsed APCs |
| Generation of Th by coincubation of APC with antigenic peptides |

specifically, the breakpoint junctions are likely neoantigens. Further, it should be possible to avoid autoimmune responses by focusing on minimal peptides corresponding to the sequences which span the breakpoint, since these would not be present in normal cells. This hypothesis was tested in animal models using synthetic peptides corresponding to the breakpoint junctions in ES and ARMS as immunogens. In these studies, peptide-pulsed APC administered intravenously, generated $CD8^+$ CTL responses capable of lysing peptide-pulsed tumor cells *in vitro* as well as tumor cells transfected to express the full-length fusion protein. Furthermore, these responses were able to reduce or irradicate tumor *in vivo*. These data demonstrate that the chimeric fusion products resulting from chromosomal translocations can serve as neoantigens. Because the translocation events are tumor specific, therapies targeting the resulting fusion proteins would be highly specific and potentially less toxic. Clinical trials are currently underway in patients with ES and ARMS to evaluate the generation of anti-tumor responses using a similar approach. In addition, studies are ongoing to not only identify additional TAA, but also to gain an understanding as to which TAA may serve as tumor rejection antigens. Since it is clear that the immune system does not react against all possible antigenic determinants, characterization of the immunodominant peptides in the tumor regression antigens will further aid in the development of effective treatments.[94]

The identification of TAA and the cloning of the genes which encode them provides numerous opportunities for the development of cancer therapies (Table 2). Therapies could utilize the TAA protein either alone or with adjuvants. Alternatively, the administration of peptides derived from the TAA protein administered alone, with adjuvants or in combination with helper peptides, has certain advantages in that this approach has been demonstrated to generate T cell responses while having minimal risk in the induction of unwanted and potentially dangerous autoimmune reactions. Anti-tumor responses generated by peptide vaccination may be augmented by manipulation of the route/mode of administration. The cloning of genes encoding TAA will facilitate their expression in high-efficiency expression systems, such as recombinant viruses or bacteria. These vectors can be engineered to express the TAA alone or in conjunction with cytokine genes or genes encoding costimulatory molecules. Furthermore, direct injection into muscle of DNA encoding antigens or the use of 'gene guns' in which DNA is attached to small particles that are mechanically propelled into cells is also an effective method of inducing immune responses.[95–100]

Anti-tumor responses have been generated by *in vitro* sensitization of peripheral blood lymphocytes (PBL) to peptide-pulsed APC or irradiated tumor cells. Repeated in vitro sensitization using immunodominant peptides from melanoma antigens pulsed onto autologous peripheral blood mononuclear cells in the presence of IL-2 resulted in the expansion of CTL (10,000-fold) over a 6-week period. Cells generated by this approach showed immune reactivity 50–100 times greater than corresponding tumor infiltrating lymphocytes (TIL)[101] and specifically recognized the appropriate immunodominant peptide as well as tumor cells as measured by lysis and cytokine release. Studies in experimental animal models suggest that specific tumor recognition as determined by lysis and cytokine secretion assays correlated highly with *in vivo* anti-tumor effects.[102] These correlates have also been observed in patients treated with autologous TIL.[103,104] In several other studies, T cells stimulated *in vitro* were capable of recognizing and lysing target cells pulsed with peptides known to bind to a particular MHC class I molecule; however, these same T cells were often incapable of recognizing and lysing the low levels of processed peptides expressed by tumor cells.[105] Thus, there is considerable heterogeneity in anti-tumor responses.

### Summary

The generation of chimeric transcription factors is a common consequence of chromosomal translocations in solid tumors. The resulting fusion proteins have been shown, in several cases, to have transforming activity. Chimeric oncoproteins may function through several mechanisms. First, a strong activation domain from one gene may be fused to the DNA-binding specificity region of another gene, leading to dysregulated expression of target genes. The fusion proteins associated with MMSP, ARMS, and PNETs are examples of this mechanism. However, in myxoid liposarcoma, the FUS/CHOP gene product appears to mediate its effect on transcription through protein–protein interactions and may not require DNA-binding. Second, a fusion partner may contribute more than an activation domain. For example, the EWS/FLI1 fusion protein of ES seems to combine the transactivation domain of EWS with the DNA-binding region of FLI1. However, the fusion protein appears to mediate novel protein–protein/protein–nucleic acid interactions. Also, the chimeric oncoprotein may heterodimerize with other transcription factors. For example, the heterodimerization of TLS/CHOP with C/EBP with C/EBP family members regulates adipocyte growth in a dominant-negative manner. Finally, chimeric genes may be overexpressed as a result of a strong promoter region from one of the partner genes. However, this mechanism has not been observed in solid tumors, but may be relevant in hematopoietic malignancies. Nonetheless, it is likely that expression of hybrid proteins in solid tumors dysregulates the transcription of key growth control genes or pathways, thereby promoting tumorigenesis.

While fusion proteins are likely to invoke a combination of the aforementioned mechanisms, the redundancy of their role in oncogenesis is noteworthy. The multiple interchange of functional domains from related genes such as *FLI1* and *ERG* in PNETs, *PAX3* and *PAX7* in ARMS and *SSX1* and *SSX2* in SS result in similar tumor phenotypes.[78] Domain-swap experiments involving EWS for TLS in TLS/CHOP showed that substitutions can be made with little change in morphology. However, other experiments in which FLI1 was exchanged for CHOP in fusions with TLS or EWS had an effect on cell morphology, such that the morphology in some cases was dependent on the DNA-binding region of the chimeric transcription factor. Finally, of note is the early onset of many of these tumors. This suggests that the genes involved in sarcoma-associated translocations have specific patterns of developmental regulation, and that dysregulation of this temporal regulation has profound effects.

Attempts at developing new therapeutic approaches to the treatment of these tumors have included immunotherapy. However, successful immunotherapeutic stratagies must meet several criteria, the first of which is the expression of TAA that are recognized by T lymphocytes. In the case of the sarcomas presented in this review, the chimeric transcription factors represent potential TAA. Studies in experimental animals suggest that the translocation breakpoints in ES and ARMS represent neoantigens which can be recognized by CTL. Furthermore, these response were sufficient to mediate *in vivo* tumor regression in animal models. Clinical vaccine studies are ongoing to evaluate the ability of these TAA to serve as tumor regression antigens. Finally, identification of the immunodominant epitopes in tumor regression antigens will favor the induction of effective anti-tumor responses. Screening vaccines and various delivery systems (peptides or proteins in adjuvants or on dendritic cells, DNA, viruses) in animals, such as HLA-transgenics, will help to identify the most promising vaccines for use in clinical trials.

### References

1 Rabbitts TH. Chromosomal translocations in human cancer. *Nature* 1994; 372:143–9.
2 Delattre O, Zucman J, Melot T, *et al.* The ewing family of tumors—a subgroup of small-round-cell tumors defined by specific chimeric transcripts. *New Engl J Med* 1994; 331:294–9.
3 Giovannini M, Biegel JA, Serra M, *et al.* EWS-*erg* and EWS-Fli1 fusion transcripts in ewing's sarcoma and primitive neuroectodermal tumors with variant translocations. *J Clin Invest* 1994; 94:489–96.
4 Downing JR, Head DR, Parham DM, *et al.* Detection of the (11;22)(q24;q12) translocation of Ewing's Sarcoma and peripheral neuroectodermal tumor by reverse transcription polymerase chain reaction. *Am J Pathol* 1993; 143:1294–1300.
5 Downing JR, Khandekar A, Shurtleff SA, *et al.* Multiplex RT-PCR assay for the differential diagnosis of alveolar rhabdomyosarcoma and Ewing's sarcoma. *Am J Pathol* 1995; 146:626–34.
6 Kawai A, Woodruff J, Healey JH, *et al.* SYT-SSX gene fusion as a determinant of morphology and prognosis in synovial sarcoma. *New Engl J Med* 1998; 338:153–60.
7 Lessnick SL, Braun BS, Denny CT, *et al.* Multiple domains mediate transformation by the Ewing's sarcoma *EWS/FLI-1* fusion gene. *Oncogene* 1995; 10:423–31.
8 May WA, Gishizky ML, Lessnick SL, *et al.* Ewing sarcoma 11;22 translocation produces a chimeric transcription factor that requires the DNA-binding domain encoded by *FLI1* for transformation. *Proc Natl Acad Sci U S A* 1993; 90:5752–6.
9 May WA, Lessnick SL, Braun BS, *et al.* The Ewing's sarcoma EWS/FLI-1 fusion gene encodes a more potent transcriptional activator and is a more powerful transforming gene than FLI-1. *Mol Cell Biol* 1993; 13:7393–8.
10 Scheidler S, Fredericks WJ, Rauscher FJ III, *et al.* The hybrid PAX3-FKHR fusion protein of alveolar rhabdomyosarcoma transforms fibroblasts in culture. *Proc Natl Acad Sci U S A* 1996; 93:9805–9.
11 Maulbecker CC, Gruss P. The oncogenic potential of Pax genes. *EMBO J* 1993; 12:2361–7.
12 Ambros IM, Ambros PF, Strehl S, *et al.* MIC2 is a specific marker for Ewing's sarcoma and peripheral primitive neuroectodermal tumors. *Cancer* 1991; 67:1886–93.
13 Fellinger EJ, Garin-Chesa P, Triche TJ, *et al.* Immunohistochemical analysis of Ewing's sarcoma cell surface antigen p 30/32MIC2. *Am J Pathol* 1991; 139:317–25.
14 Turc-Carel C, Philip I, Berger MP, *et al.* Chromosomal translocations in ewing's sarcoma. *New Engl J Med* 1983; 309:497–8.
15 Aurias A, Rimbaut C, Buffe D, *et al.* Chromosomal translocations in Ewing's sarcoma. *New Engl J Med* 1983; 309:496–7.
16 Whang-Peng J, Triche TJ, Knutsen T, *et al.* Chromosome translocation in peripheral neuroepithelioma. *New Engl J Med* 1984; 311:584–5.
17 Turc-Carel C, Aurias A, Mugneret F, *et al.* Chromosomes in Ewing's sarcoma: An evaluation of 85 cases and remarkable consistency of t(11;22)(q24;q12). *Cancer Genet Cytogenet* 1988; 32:229–38.
18 Triche TJ. Pathology of pediatric malignancies. In: Pizzo PA, Poplack DG, eds. Principles and practice of pediatric oncology. 2nd ed. Philadelphia: J.B. Lippincott, 1993:115–52.
19 Delattre O, Zucman J, Plougastel B, *et al.* Gene fusion with an ETS DNA-binding domain caused by chromosome translocation in human tumours. *Nature* 1992; 359:162–5.
20 Mao X, Miesfeldt S, Yang H, *et al.* The FLI-1 and chimeric EWS-FLI-1 oncoproteins display similar DNA binding specificities. *J Biol Chem* 1994; 269:18216–22.
21 Ohno T, Ouchida M, Lee L, *et al.* The EWS gene, involved in Ewing family of tumors, malignant melanoma of soft parts and desmoplastic small round cell tumors, codes for an RNA binding protein with novel regulatory domains. *Oncogene* 1994; 9:3087–3097.
22 Aman P, Panagopoulos I, Lassen C, *et al.* Expression patterns of the human sarcoma-associated genes FUS and EWS and the genomic structure of FUS. *Genomics* 1996; 37:1–8.
23 Zucman J, Delattre O, Desmaze C, *et al.* Cloning and characterization of the Ewing's sarcoma and peripheral neuroepithelioma t(11;22) translocation breakpoints. *Genes Chromosomes Cancer* 1992; 90:271–7.
24 Plougastel B, Zucman J, Peter M, *et al.* Genomic structure of the EWS gene and its relationship to EWSR1, a site of tumor-associated chromosome translocation. *Genomics* 1993; 18:609–15.
25 Ben-David Y, Giddens EB, Letwin K, *et al.* Erythroleukemia induction by Friend murine leukemia virus: Insertional activation of a new member of the ets family, Fli 1. *Genes Dev* 1991; 5:908–18.
26 Wasylyk B, Hahn SL, Giovane A. The Ets family of transcription factors. *Eur J Biochem* 1993; 211:7–18.
27 Klemsz MJ, Maki RA, Papyannopoulou T, Characterization of the ets oncogene famly member, Fli-1. *J Biol Chem* 1993; 268:5769–73.
28 Zucman J, Melot T, Desmaze C, *et al.* Combinatorial generation of variable fusion protiens in the Ewing family of tumours. *EMBO J* 1993; 12:4481–7.
29 Bailly RA, Bosselut R, Zucman J, *et al.* DNA-binding and transcriptional activation properties of the EWS-FLI-1 fusion protein resulting from the t(11;22) translocation in Ewing sarcoma. *Mol Cell Biol* 1994; 14:3230–41.
30 Ohno T, Rao VN, Reddy SP. EWS/Fli-1 chimeric protein is a transcriptional activator. *Cancer Res* 1993; 53:5859–63.
31 Magnaghi-Jaulin L, Masutani H, Robin P, *et al.* SRE elements are binding sites for the fusion protein EWS-FLI-1. *Nucleic Acids Res* 1996; 24:1052–8.
32 Braun BS, Frieden R, Lessnick SL, *et al.* Identification of target genes for the Ewing's sarcoma EWS/FLI fusion protein by representational difference analysis. *Mol Cell Biol* 1995; 15:4623–30.
33 Kovar H, Aryee DN, Jug G, *et al.* EWS/FLI-1 antagonists induce growth inhibition of Ewing tumor cells in vitro. *Cell Growth Diff* 1996; 7:429–437.
34 Tanaka K, Iwakuma T, Harimaya K, *et al.* EWS-Fli1 antisense oligodeoxynucleotide inhibits proliferation of human Ewing's sarcoma and primitive neuroectodermal tumor cells. *J Clin Invest* 1997; 99:239–247.
35 Yi H, Fujimura Y, Ouchida M, *et al.* Inhibition of apoptosis by normal and aberrant Fli-1 and erg proteins involved in human soldi tumors and leukemias. *Oncogene* 1997; 14:1259–68.
36 Sorensen PHB, Lessnick SL, Lopez-Terrada D, *et al.* A second Ewing's sarcoma translocation, t(21;22), fuses the EWS gene to another ETS-family transcription factor, ERG. *Nat Genet* 1994; 6:146–51.
37 Jeon I, Davis JN, Braun BS, *et al.* A variant Ewing's sarcoma translocation t(7;22) fuses the *EWS* gene to the ETS gene *ETV1*. *Oncogene* 1995; 10:1229–34.
38 Peter M, Couturier J, Pacquement H, *et al.* A new member of the ETS family fused to EWS in Ewing tumors. *Oncogene* 1997; 14:1159–64.
39 Turc-Carel C, Lizard-Nacol S, Justrabo E, *et al.* Consistent chromosomal translocation in alveolar rhabdomyosarcoma. *Cancer Genet Cytogenet* 1986; 19:361–2.
40 Barr FG, Galili N, Holick J, *et al.* Rearrangement of the PAX3 paired box gene in the paediatric solid tumour alveolar rhabdomyosarcoma. *Nat Genet* 1993; 3:113–17.

41 Galili N, Davis RJ, Fredericks WJ, *et al.* Fusion of a fork head domain gene to *PAX3* in the solid tumour alveolar rhabdomyosarcoma. *Nat Genet* 1993; 5:230–5.
42 Shapiro DN, Sublett JE, Li B, *et al.* Fusion of *PAX3* to a member of the forkhead family of transcription factors in human alveolar rhabdomyosarcoma. *Cancer Res* 1993; 53:5108–12.
43 Biegel JA, Nycum LM, Valentine V, *et al.* Detection of the t(2;13)(q35;q14) and PAX3-FKHR fusion in alveolar rhabdomyosarcoma by fluorescence in situ hybridization. *Gene Chromosones Cancer* 1995; 12:186–92.
44 Mansouri A, Hallonet M, Gruss P. Pax genes and their roles in cell differentiation and development. *Curr Opin Cell Biol* 1996; 8:851–7.
45 Macina RA, Barr FG, Galili N, *et al.* Genomic organization of the human PAX3 gene: DNA sequence analysis of the region disrupted in alveolar rhabdomyosarcoma. *Genomics* 1995; 26:1–8.
46 Fredericks WJ, Galili N, Mukhopadhyay S, *et al.* The PAX3-FKHR fusion protein created by the t(2;13) translocation in alveolar rhabdomyosarcomas is a more potent transcriptional activator than PAX3. *Mol Cell Biol* 1995; 15:1522–35.
47 Bennicelli JL, Fredericks WJ, Wilson RB, *et al.* Wild type PAX3 protein and the PAX3-FKHR fusion protein of alveolar rhabdomyosarcoma contain potent, structurally distinct transcriptional activation domains. *Oncogene* 1995; 11:119–30.
48 Bennicelli JL, Edwards RH, Barr FG. Mechanism for transcriptional gain of function resulting from chromosomal translocation in alveolar rhabdomyosarcoma. *Proc Natl Acad Sci USA* 1996; 93:5455–9.
49 Sublett JE, Jeon IS, Shapiro DN. The alveolar rhabdomyosarcoma PAX3/FKHR fusion protein is a transcriptional activator. *Oncogene* 1995; 11:545–52.
50 Davis RJ, Barr FG. Fusion genes resulting from alternative chromosomal translocations are overexpressed by gene-specific mechanisms in alveolar rhabdomyosarcoma. *Proc Natl Acad Sci USA* 1997; 94:8047–51.
51 Bernasconi M, Remppis A, Fredericks WJ, *et al.* Induction of apoptosis in rhabdomyosarcoma cells through down-regulation of PAX proteins. *Proc Natl Acad Sci USA* 1996; 93:13164–9.
52 Davis RJ, D'Cruz CM, Lovell MA, *et al.* Fusion of PAX7 to FKHR by the variant t(1;13)(p36;q14) translocation in alveolar rhabdomyosarcoma. *Cancer Res* 1994; 54:2869–72.
53 Hicks MJ, Saldivar VA, Chintagumpala MM, *et al.* Malignant melanoma of soft parts involving the head and neck region: review of literature and case report. *Ultrastruct Pathol* 1995; 19:395–400.
54 Chung EB, Enzinger FM. Malignant melanoma of soft parts. *Am J Surg Pathol* 1983; 7:405–13.
55 Bridge JA, Sreekantaiah C, Neff JR, *et al.* Cytogenetic findings in clear cell sarcoma of tendons and aponeuroses. *Cancer Genet Cytogenet* 1991; 52:101–6.
56 Stenman G, Kindblom L-G, Angervall L. Reciprocal translocation t(12;22)(q13;q13) in clear cell sarcoma of tendons and aponeuroses. *Genes Chromosones Cancer* 1992; 4:122–7.
57 Hai T, Liu F, Coukos WJ, *et al.* Transcription factor ATF cDNA clones: An extensive family of leucine zipper proteins able to selectively form DNA-binding heterodimers. *Genes Dev* 1989; 3:2083–90.
58 Zucman J, Delattre O, Desmaze C, *et al.* *EWS* and *ATF-1* gene fusion induced by t(12;22) translocation in malignant melanoma of soft parts. *Nat Genet* 1993; 4:341–5.
59 Speleman F, Delattre O, Peter M, *et al.* Malignant melanoma of the soft parts (clear cell sarcoma): confirmation of EWS and ATF-1 gene fusion caused by a t(12;22) translocation. *Mod Pathol* 1997; 10:496–9.
60 Brown AD, Lopez-Terrada D, Denny C, *et al.* Promoters containing ATF-binding sites are de-regulated in cells that express the EWS/ATF1 oncogene. *Oncogene* 1995; 10:1749–56.
61 Gerald WL, Miller HK, Battifora H, *et al.* Intra-abdominal desmoplastic small round-cell tumor. Report of 19 cases of a distinctive type of high-grade polyphenotypic malignancy affecting young individuals. *Am J Surg Pathol* 1991; 15:499–513.
62 Sawyer JR, Tryka AF, Lewis JM. A novel reciprocal chromosome translocation t(11;22)(p13;q12) in an intraabdominal desmoplastic small round-cell tumor. *Am J Surg Pathol* 1992; 16:411–6.
63 Shen WP, Towne B, Zadeh TM. Cytogenetic abnormalities in an intra-abdominal desmoplastic small cell tumor. *Cancer Genet Cytogenet* 1992; 64:189–91.
64 Benjamin LE, Fredericks WJ, Barr FG, *et al.* Fusion of the EWS1 and WT1 genes as a result of the t(11;22)(p13;q12) translocation in desmolplastic small round cell tumors. *Med Pediatr Oncol* 1996; 27:434–9.
65 Ladanyi M, Gerald W. Fusion of the *EWS* and *WT1* genes in the desmoplastic small round cell tumor. *Cancer Res* 1994; 54:2837–40.
66 Gerald WL, Rosai J, Ladanyi M. Characterization of the genomic breakpoint and chimeric transcripts in the EWS-WT1 gene fusion of desmoplastic small round cell tumor. *Proc Natl Acad Sci U S A* 1995; 92:1028–32.
67 Rauscher FJ III. Chromosome translocation-mediated conversion of a tumor suppressor gene into a dominant oncogene: fusion of EWS1 to WT1 in demmoplastic small round cell tumors. *Curr Top Microbiol Immunol* 1997; 220:151–62.
68 Dal Cin P, Sciot R, Panagopoulos I, *et al.* Additional evidence of a variant translocation t(12;22) with EWS/CHOP fusion in myxoid liposarcoma. *J Pathol* 1997; 182:437–41.
69 de Bruijn DR, Baats E, Zechner U, *et al.* Isolation and characterization of the mouse homolog of SYT, a gene implicated in the development of human synovial sarcomas. *Oncogene* 1996; 13:643–48.
70 Clark J, Rocques PJ, Crew AJ, *et al.* Identification of novel genes, SYT and SSX, involved in the t(X:18)(p11.2;q11.2) translocation found in human synovial sarcoma. *Nat Genet* 1994; 7:502–8.
71 Crew AJ, Clark J, Fisher C, *et al.* Fusion of SYT to two genes, SSX1 and SSX2, encoding proteins with homology to the Kruppel-associated box in human synovial sarcoma. *EMBO J* 1995; 14:2333–40.
72 Licht JD, Ro M, English MA, *et al.* Selective repression of transcriptional activators at a distance by the Drosophila Kruppel protein. *Proc Natl Acad Sci USA* 1993; 90:11361–5.
73 Margolin JF, Friedman JR, Meyer WK, *et al.* Kruppel-associated boxes are potent transcriptional repression domains. *Proc Natl Acad Sci USA* 1994; 91:4509–13.
74 de Leeuw B, Balemans M, Geurts van Kessel A. A novel Kruppel-box containing the SSX gene (SSX3) on the human X chromosome is not implicated in t(X;18)-positive synovial sarcomas. *Cytogenet Cell Genet* 1996; 73:179–83.
75 Crozat A, Åman P, Mandahl N, *et al.* Fusion of CHOP to a novel RNA-binding protein in human myxoid liposarcoma. Nature 1993; 363:640–4.
76 Rabbitts TH, Forster A, Larson R, *et al.* Fusion of the dominant negative transcription regulator CHOP

with a novel gene FUS by translocation t(12;16) in malignant liposarcoma. *Nat Genet* 1993; 4:175–80.

77 Panagopoulos I, Mandahl N, Ron D, *et al.* Characterization of the CHOP breakpoints and fusion transcripts in myxoid liposarcomas with the 12;16 translocation. *Cancer Res* 1994; 54:6500–3.

78 Zinszner H, Albalat R, Ron D. A novel effector domain from the RNA-binding protein TLS or EWS is required for oncogenic transformation by CHOP. *Genes Dev* 1994; 8:2513–26.

79 Barone MV, Crozat A, Tabaee A, *et al.* CHOP (GADD153) and its oncogenic variant, TLS-CHOP, have opposing effects on the induction of G1/S arrest. *Genes Dev* 1994; 8:453–64.

80 Ron D, Habener JF. CHOP, a novel developmentally regulated nuclear protein that dimerizes with transcription factors C/EBP and LAP and functions as a dominant-negative inhibitor of gene transcription. *Genes Dev* 1992; 6:439–53.

81 Germain RN, Margulies DH. The biochemistry and cell biology of antigen processing and presentation. *Annu Rev Immunol* 1993; 11:403–50.

82 Townsend A, Bodmer H. Antigen recognition by class I-restricted T lymphocytes. *Annu Rev Immunol* 1989; 7:601–24.

83 Houbiers JGA, Nijman HW, van der Burg SH, *et al.* In vitro induction of human cytotoxic T lymphocyte responses against peptides of mutant and wild-type p53. *Eur J Immunol* 1993; 23:2072–7.

84 Carbone DP, Ciernik IF, Yanuck M, *et al.* Mutant p53 and ras proteins as immunotherapeutic targets. *Ann Oncol* 1994; 5:117.

85 Yanuck M, Carbone DP, Pendleton CD, *et al.* A mutant p53 tumor suppressor protein is a target for peptide-induced $CD^{8+}$ cytotoxic T cells. *Cancer Res* 1993; 53:3257–61.

86 Theobald M, Biggs J, Dittmer D, *et al.* Targeting p53 as a general tumor antigen. *Proc Natl Acad Sci U S A* 1995; 92:11993–7.

87 Noguchi Y, Richards EC, Chen Y-T, *et al.* Influence of interleukin 12 on p53 peptide vaccination against established Meth A sarcoma. *Proc Natl Acad Sci USA* 1995; 92:2219–23.

88 Mayordomo JI, Loftus DJ, Sakamoto H, *et al.* Therapy of murine tumors with p53 wild-type and mutant sequence peptide-based vaccines. *J Exp Med* 1996; 183:1357–65.

89 Nanda NK, Sercarz EE. Induction of anti-self-immunity to cure cancer. *Cell* 1995; 82:13–8.

90 Kawakami Y, Eliyahu S, Delgado CH, *et al.* Cloning of the gene coding for a shared human melanoma antigen recognized by autologous T cells infiltrating into tumor. *Proc Natl Acad Sci USA* 1994; 91:3515–9.

91 Robbins PF, El-Gamil M, Li YF, *et al.* A mutated b-catenin gene encodes a melanoma-specific antigen recognized by tumor infiltrating lymphocytes. *J Exp Med* 1996; 183:1185–92.

92 Cox AL, Skipper J, Chen Y, *et al.* Identification of a peptide recognized by five melanoma-specific human cytotoxic T cell lines. *Science* 1994; 264:716–9.

93 Van der Bruggen P, Traversari C, Chomez P, *et al.* A gene encoding an antigen recognized by cytolytic T lymphocytes on a human melanoma. *Science* 1991; 254: 643–7.

94 Sercarz EE, Lehmann PV, Ametani A, *et al.* Dominance and crypticity of T cell antigenic determinants. *Annu Rev Immunol* 1993; 11:729–66.

95 Tang D, DeVit M, Johnston SA. Genetic immunization is a simple method for eliciting an immune response. *Nature* 1992; 356: 152–4.

96 Torres CAT, Iwasaki A, Barber BH, *et al.* Differential dependence on target site tissue for gene gun and intramuscular DNA immunizations. *J Immunol* 1997; 158:4529–32.

97 Wang B, Ugen KE, Srikantan V, *et al.* Genetic Immunization: A novel method for vaccine development against HIV. In: Ginsberg HS, Brown F, Chanock RM, Lerner RA, eds. Vaccines 93: Modern approaches to new vaccines including the prevention of AIDS. Cold Spring Harbor: Cold Spring Harbor Laboratory Press, 1993:143–50.

98 Irvine KR, Rao JB, Rosenberg SA, *et al.* Cytokine enhancement of DNA immunization leads to effective treatment of established pulmonary metastases. *J Immunol* 1996; 156:238–45.

99 Doe B, Selby M, Barnett S, *et al.* Induction of cytotoxic T lymphocytes by intramuscular immunization with plasmid DNA is facilitated by bone marrow-derived cells. *Proc Natl Acad Sci U S A* 1996; 93:8578–83.

100 Plautz GE, Yang ZY, Wu BY, *et al.* Immunotherapy of malignancy by *in vivo* gene transfer into tumors. *Proc Natl Acad Sci USA* 1993; 90:4645–9.

101 Parkhurst MR, Salgaller ML, Southwood S, *et al.* Improved induction of melanoma-reactive CTL with peptides from the melanoma antigen gp 100 modified at HLA-A*0201-binding residues. *J Immunol* 1996; 157:2539–48.

102 Barth RJ, Mule JJ, Spiess PJ, *et al.* Interferon gamma and tumor necrosis factor have a role in tumor regressions mediated by murine CD8+ tumor-infiltrating lymphocytes. *J Exp Med* 1991; 173:647–58.

103 Rosenberg SA, Yannelli JR, Yang JC, *et al.* Treatment of patients with metastatic melanoma with autologous tumor-infiltrating lymphocytes and interleukin 2. *J Natl Cancer Inst* 1994; 86:1159–66.

104 Schwartzentruber DJ, Hom SS, Dadmarz R, *et al.* In vitro predictors of therapeutic response in melanoma patients receiving tumor-infiltrating lyumphocytes and interleukin-2. *J Clin Oncol* 1994; 12:1475–83.

105 Van Elas A, Nijman HW, Van Der Minne CE, *et al.* Induction and characterization of cytotoxic T-lymphocytes recognizing a mutated p21 RAS peptide presented by HLA-A2010. *Int J Cancer* 1995; 61:389–96.
